# Research on Plant RNA-Binding Protein Prediction Method Based on Improved Ensemble Learning

**DOI:** 10.3390/biology14060672

**Published:** 2025-06-10

**Authors:** Hongwei Zhang, Yan Shi, Yapeng Wang, Xu Yang, Kefeng Li, Sio-Kei Im, Yu Han

**Affiliations:** 1 Faculty of Applied Sciences, Macao Polytechnic University, Macau 999078, China; hongwei.zhang@mpu.edu.mo (H.Z.); xuyang@mpu.edu.mo (X.Y.); marcusim@mpu.edu.mo (S.-K.I.); 2 State Key Laboratory of Networking and Switching Technology, Beijing University of Posts and Telecommunications, Beijing 100876, China; 3 Center for Artificial Intelligence Driven Drug Discovery, Faculty of Applied Sciences, Macao Polytechnic University, Macau 999078, China; kefengl@mpu.edu.mo; 4 Faculty of Civil Engineering, Southwest Forestry University, Kunming 650224, China; hanyu@swfu.edu.cn

**Keywords:** plant, RNA-binding proteins, RBPs, TextCNN, ensemble learning

## Abstract

(1) RNA-binding proteins (RBPs) play a crucial role in regulating gene expression in plants, affecting growth, development, and stress responses. Accurate prediction of plant-specific RBPs is vital for understanding gene regulation and enhancing genetic improvement. (2) Methods: We propose an ensemble learning method that integrates shallow and deep learning. It integrates prediction results from SVM, LR, LDA, and LightGBM into an enhanced TextCNN, using K-Peptide Composition (KPC) encoding (k = 1, 2) to form a 420-dimensional feature vector, extended to 424 dimensions by including those four prediction outputs. Redundancy is minimized using a Pearson correlation threshold of 0.80. (3) Results: On the benchmark dataset of 4992 sequences, our method achieved an ACC of 97.20% and 97.06% under 5-fold and 10-fold cross-validation, respectively. On an independent dataset of 1086 sequences, our method attained an ACC of 99.72%, an ${F1}_{score}$ of 99.72%, an MCC of 99.45%, an SN of 99.63%, and an SP of 99.82%, outperforming RBPLight by 12.98 percentage points in ACC and the original TextCNN by 25.23 percentage points. (4) Conclusions: These results highlight our method’s superior accuracy and efficiency over PSSM-based approaches, enabling large-scale plant RBP prediction.

## Simple Summary

Plants rely on special proteins called RNA-binding proteins, to control their genes, guiding their growth and development. Identifying these proteins is challenging and slowing down plant research. Our research proposes an effective computational method to find these proteins by studying their patterns, like decoding a puzzle. We merged various learning techniques to study 4992 plant proteins, achieving an impressive 97.20% accuracy in tests, and even hit 99.72% on a separate set of 1086 proteins, surpassing other methods. Our method accurately identifies RNA-binding proteins that control plant gene, making it easier to study how plants grow and develop. This useful tool helps researchers explore plant biology, advancing research into plant genetics. By improving our understanding of gene regulation, our work supports discoveries that benefit plant science.

## 1. Introduction

RNA-binding proteins (RBPs) [1] are a class of proteins that regulate the biological functions of RNA by binding to RNA molecules. They regulate RNA stability, transcription rates, and RNA splicing, thereby influencing gene expression levels and patterns [2]. For example, they contribute to transcriptional regulation, RNA splicing, RNA modification, and RNA transport and localization [3]. Recent studies have confirmed the widespread presence of RBPs in humans, animals, and plants [4]. Their abnormal expression or mutations in RBPs are directly linked to the onset and progression of various diseases. For instance, mutations in specific RBPs have been found to be associated with human neurological disorders, metabolic disorders, and tumors [5]. Consequently, RBPs have become crucial targets for disease research [6] and drug development [7]. In plants, they play a vital role in growth and development, environmental adaptation, and stress response [8]. Further research will enhance our understanding of plant gene regulation mechanisms and provide a theoretical foundation for genetic improvement and stress-resistant breeding in plants [9]. This study proposes a novel ensemble learning framework that integrates shallow and deep learning approaches to develop an efficient computational tool for accurately predicting plant-specific RBPs. This tool overcomes the computational bottlenecks of traditional methods reliant on complex evolutionary features, such as position-specific scoring matrices (PSSM). Through carefully designed feature selection strategies and model architecture, it demonstrates superior performance and generalization ability on cross-validation and independent dataset, providing valuable support for advancing research on RBP functions in plant growth, development, and environmental adaptation.

In recent years, the application of machine learning methods for predicting RBPs has emerged as a prominent research area [10]. With the capability of machine learning to analyze and process data on a large scale, it becomes feasible to employ these methods for large-scale prediction of RBPs [11]. Utilizing the prediction results as guidance for subsequent laboratory identification and confirmation can markedly enhance prediction efficiency and save substantial time [12,13]. This approach is gaining increasing acceptance among researchers [14]. This study focuses on the prediction method for RBPs in plants. A prediction model for RBPs is constructed based on a deep machine learning method, and a rigorous experimental process is designed to validate the efficacy of the proposed method [15].

RBPs are essential regulators of gene expression in plants, modulating RNA stability, transcription, and splicing to influence growth, development, and stress responses. Accurate prediction of plant-specific RBPs is critical for elucidating gene regulation mechanisms and supporting genetic improvement efforts. Recent advances in machine learning have significantly enhanced RBP prediction, leveraging both shallow and deep learning techniques to analyze protein sequences [16,17].

Early approaches relied on shallow learning methods for their interpretability and efficiency. For instance, Zhang et al. [18] developed RBPPred using Support Vector Machine (SVM) [19] trained on features such as amino acid composition, dihedral angles, physicochemical properties, and sequence conservation. Under 10-fold cross-validation, RBPPred achieved 83% accuracy for 2780 RBPs and 96% for 7093 non-RBPs (MCC = 0.808), with 84% sensitivity and 97% specificity on a human proteome test set. Ensemble learning methods have further improved predictive power by integrating multiple learners. Mishra et al. [20] proposed AIRBP, which combines evolutionary information, physicochemical properties, and disorder features, achieving 95.84% accuracy and an MCC of 0.899 under 10-fold cross-validation, with robust performance on yeast and Arabidopsis datasets. Concurrently, deep learning has emerged as a powerful tool for RBP recognition. Niu et al. [21] introduced rBPDL, a convolutional neural network (CNN) [22] and long short-term memory (LSTM) [23] enhanced with ensemble learning, yielding macro Area Under Curve (AUC), micro AUC, and weighted AUC values of 0.936, 0.962, and 0.946, respectively, on the RBP68 dataset. Focusing on plant-specific RBPs, Pradhan et al. [24] integrated five deep learning and ten shallow learning methods with sequence and evolutionary features, achieving an AUC of 91.24% and AU-PRC of 91.91% via Light Gradient Boosting Machine (LightGBM) [25] under 5-fold cross-validation, improving to 94.00% and 94.50% on independent dataset. In another research, for prokaryotic RBPs, Pradhan et al. [26] proposed RBProkCNN, using CNNs with position-specific scoring matrix (PSSM) derived features selected by XGB Variable Importance Measures (XGB-VIM) [27] and LightGBM Variable Importance Measures (LGBM-VIM), attaining 98.04% AUC and 98.19% AU-PRC.

Despite these advances, challenges remain, including feature redundancy, reliance on computationally intensive evolutionary features, such as PSSM [28], and limited generalization for plant-specific RBPs. These challenges highlight the need for more efficient feature extraction methods that balance predictive power and computational cost, particularly for plant-specific RBPs where sequence-based approaches often outperform structure-based methods [29,30]. This study overcomes the key challenges in plant-specific RBP prediction—namely, feature redundancy, reliance on computationally intensive evolutionary features like PSSM, and limited generalization—by introducing an improved ensemble learning framework. This framework mitigates feature redundancy through a Pearson correlation-based selection strategy (threshold of 0.80), reduces computational cost by replacing PSSM with lightweight KPC encoding (k = 1, 2), and enhances generalization by integrating diverse machine learning methods: SVM, Logistic Regression (LR) [31], Linear Discriminant Analysis (LDA) [32] and LightGBM [33] to capture linear patterns and an improved TextCNN to extract complex sequence motifs, ensuring robust performance across varied plant-specific datasets. We hypothesize that this framework, by leveraging lightweight KPC encoding and rigorous feature selection, will significantly improve the accuracy, stability, and computational efficiency of plant-specific RBP prediction, outperforming existing PSSM-based methods.

The structure of this paper is as follows. The first section is the introduction, which primarily discusses the important role of RBPs in plants, the progress of existing prediction methods, and the associated challenges. The second section covers the materials and methods, which primarily detail the dataset, KPC coding method, shallow and ensemble learning methods, improved TextCNN model, attention mechanism, and evaluation metrics. The third section presents the results and discussion, analyzing the model’s performance on the benchmark dataset and independent dataset in detail, discussing the influence of various feature combinations, and comparing it with existing advanced methods. And summarizing the advantages and limitations of the method. Finally, the fourth section is the conclusion, which emphasizes the method’s advantages and proposes future research directions.

## 2. Materials and Methods

This study utilized datasets from existing research and proposed an improved ensemble learning method for predicting RBPs in plants. This method relies solely on a simple sequence-based protein representation but implements a prediction model with efficient performance.

### 2.1. The Overall Framework of the Prediction Method

**Figure 1:**
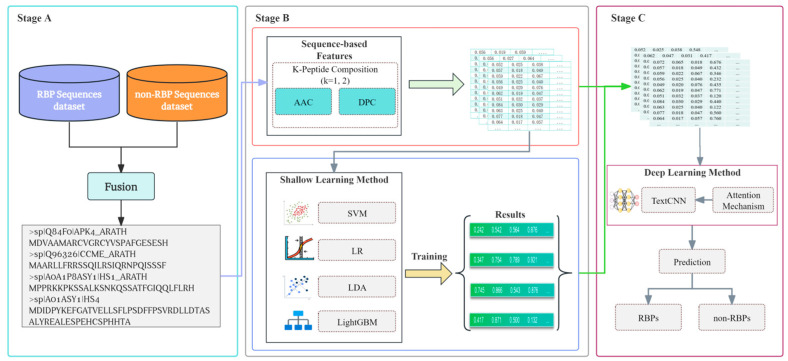
Shows the overall structure of the prediction framework of this study. First, RBPs and non-RBP sequence data are collected in (**Stage A**), and then the protein sequence is first encoded in (**Stage B**). This study introduces the protein sequence-based AAC, TPC, and DPC methods for sequence encoding. After encoding, machine learning methods, such as SVM, LR, LDA, and LightGBM, are applied for training and prediction. Finally, in (**Stage C**), the encoded protein sequence and the predicted results of the four machine learning methods are fused; then, a deep learning method (TextCNN) is used for higher-dimensional feature abstraction, and finally, classification prediction is performed to classify RBPs from non-RBPs.

This method employs simple protein sequence features. It integrates prediction results of three shallow learning methods, SVM, LR, and LDA, and one ensemble learning method, LightGBM. Finally, it abstracts the features in a higher dimension based on the TextCNN deep learning method and predicts and verifies them on the independent dataset. The framework of the proposed prediction method is shown in Figure 1.

### 2.2. Dataset

This study used the dataset suggested by Pradhan UK et al. [24], which collected RBP sequences and non-RBP sequences from 36 plants from CISBP-RNA and UniProtKB databases. RBPs were screened using the GO term “RNA binding”, while non-RBPs were defined based on no relevant annotations. After removing redundancy using the CD-HIT tool (40% sequence similarity threshold), a balanced training set containing 2496 RBPs and 2496 non-RBPs was constructed; this dataset serves as a benchmark dataset, along with an independent dataset of 543 pairs of RBPs and non-RBPs. The dataset was downloaded from the website RBPLight on 2 December 2024.

### 2.3. Protein Sequence Encoding

Protein sequence encoding is a process of converting the amino acid sequence of a protein into a numerical or symbolic sequence that can be used for computational analysis. Typically, proteins are composed of 20 different amino acids, each with unique chemical and physical properties. The encoding method represents these amino acids with numbers, vectors, or other forms of symbols, depending on the purpose of the research and the needs of subsequent analysis. For example, a simple digital encoding can be used to assign a unique number to each amino acid, or a physicochemical property vector encoding can be used to quantify the hydrophobicity, polarity, charge, and other properties of amino acids into multidimensional vectors. Through this encoding, protein sequences can be recognized and processed by computer programs and then used in a variety of bioinformatics applications such as structure prediction, functional analysis, and evolutionary research. This study introduces a simple and efficient protein sequence encoding method. The k-peptide composition (KPC) [34] is a computational method for sequence analysis. It is commonly used in bioinformatics [35] and widely applied in protein classification and RBP prediction. This method constructs a feature vector by counting the frequency of occurrence of subsequences (k-peptides) of length k in the sequence to represent the composition information of the sequence [36]. For example, in a protein sequence “ACDEFG”, if k=3, the possible 3-peptides include “ACD”, “CDE”, “DEF”, and “EFG”. By calculating the frequency of each possible 3-peptides in the sequence, the protein sequence can be represented as a vector, which can be defined by the following Equation (1).

(1) $$\begin{array}{c} \begin{array}{c} V_{KPC}=(f_{1},{\;f}_{2},\;f_{3},\cdots ,\;f_{20^{k}})\\ f_{i}=\frac{N_{i}}{L-k+1} \end{array} \end{array}$$

where, $N_{i}$ represents the number of times k-peptides appear in the sequence, L−k+1 is the total number of k-peptides in the sequence, and $f_{i}$ represents the frequency of occurrence of the i-th k-peptides in the sequence. The dimension of the encoded sequence is $20^{k}$.

When k=1, the KPC method can be shown as Equation (2).

(2) $$\begin{array}{c} V_{KPC}=(f_{1},{\;f}_{2},\;f_{3},\cdots ,\;f_{20}) \end{array}$$

here, $N_{i}$ represents the number of times the *i*-th 1-peptide appears in the sequence, and $f_{i}$ represents the frequency of occurrence of the *i*-th amino acid in the sequence. The dimension of the encoded sequence is 20. In other words, when *k* = 1, the KPC method is equivalent to the amino acid composition (AAC) [37] method, which represents protein sequences by calculating the frequency of each amino acid. Specifically, proteins are composed of 20 standard amino acids. The AAC method calculates the number of occurrences or proportions of each amino acid in the sequence, thereby generating a 20-dimensional vector to represent the protein sequence. This method is simple and intuitive and can provide basic composition information of protein sequences. It is often used in bioinformatics research such as protein classification and function prediction.

Similarly, when k=2, the method can be expressed as the Di-Peptide Composition (DPC) [38] method, as defined in Equation (3).

(3) $$\begin{array}{c} V_{DPC}=(f_{1},{\;f}_{2},\;f_{3},\cdots ,\;f_{400}) \end{array}$$

here, $N_{i}$ represents the number of times the 2-peptides appear in the sequence, $f_{i}$ represents the frequency of the 2-peptides in the sequence, and the dimension of the encoded sequence is 400.

In the KPC method, the choice of the k value is a key factor influencing feature extraction and model performance. The k value determines the length of k-peptides, directly influencing the dimension of the feature vector and the complexity of the captured sequence patterns. If *k* is too small (such as *k* = 1), the feature vector has 20 dimensions, capturing only the frequency of a single amino acid, which provides limited information and struggles to reflect the complex patterns needed for RBP prediction. When *k* = 3, the sequence dimension increases to 8000. Although this captures longer sequence patterns, the feature vector becomes sparse, and both computational cost and noise increase significantly. Studies have shown that *k* = 2 (i.e., DPC, dimension 400) is often a better choice, balancing pattern capture ability and computational efficiency, and is particularly suitable for capturing the combinatorial information of adjacent amino acids, especially in RBP prediction tasks. The choice is further supported by studies demonstrating that DPC effectively captures local sequence patterns critical for protein/RNA interactions, enhancing the accuracy of RBP prediction methods [39,40]. This study combined two *k* values (*k* = 1, 2) and concatenated the features encoded by both k values. Therefore, after introducing the KPC method, we get a total of 420 dimensional vectors.

### 2.4. Shallow Learning Method

Shallow learning methods have been widely utilized in previous studies for identifying RBPs due to their strong interpretability and computational efficiency [41,42]. Methods such as SVM, LR, and Linear Discriminant Analysis (LDA) have demonstrated robust performance in classifying protein sequences based on simple feature representations. In this study, we leverage these methods to enhance the representational capacity of protein-encoded sequences and improve prediction accuracy for plant-specific RBPs. We evaluated multiple shallow learning methods on a benchmark dataset of 4992 sequences and selected LR, LDA, and SVM based on their theoretical strengths, high Area Under the Curve (AUC) values, and low inter-feature correlations. LR, a linear classifier, captures linear relationships in sequence features via maximum likelihood estimation, offering simplicity and interpretability (AUC = 0.804, Figure 5; Table 2 ACC = 76.68% in D2). LDA, also linear, maximizes inter-class variance to form discriminative boundaries, effective for high-dimensional sequence data (AUC = 0.804, Figure 5; Table 2: ACC = 79.41%, MCC +5.34% in D3). SVM, using an RBF kernel, excels at modeling non-linear patterns while implicitly capturing linear relationships through high-dimensional mappings, demonstrating superior performance (AUC = 0.845, Figure 5; Table 2: ACC + 12.04% in D1). These methods were chosen to balance linear and non-linear pattern capture, with their prediction outputs forming a 3-dimensional vector integrated into the 424-dimensional sequence encoding method (Section 2.3). Feature selection with a 0.80 Pearson correlation threshold ensured low inter-feature correlations, enhancing robustness (Figure 7). This approach enriches the feature set with distinct linear and discriminative patterns, providing a solid foundation for integration with ensemble (Section 2.5) and deep learning methods (Section 2.6) while maintaining computational simplicity and interpretability for downstream classification tasks.

### 2.5. Ensemble Learning Method

Ensemble learning methods, which combine multiple weak learners to achieve superior predictive performance, have also shown significant promise in RBP identification tasks. Algorithms such as Random Forest (RF) [43], Gradient Boosting Decision Tree (GBDT) [44], Extreme Gradient Boosting Tree (XGB) [45], and LightGBM are well-suited for protein sequence analysis. In this study, we evaluated these methods on a benchmark dataset of 4992 sequences and selected LightGBM as the representative ensemble method based on its theoretical strengths, high Area Under the Curve (AUC) value, and computational efficiency. LightGBM, a gradient boosting method, excels in capturing non-linear sequence dependencies through histogram-based tree splitting, while its shallow trees approximate linear patterns, complementing the linear modeling of LR and LDA (Section 2.4) and the non-linear capabilities of SVM (Section 2.4). LightGBM achieved an AUC of 0.832 (Figure 5), outperforming XGB (AUC = 0.827), which was excluded due to high correlation (Pearson = 0.98, Figure 6). Its prediction output contributes a 1-dimensional feature, forming part of the 424-dimensional sequence encoding method (Section 2.3), alongside the 3-dimensional outputs from SVM, LR, and LDA (Section 2.4). Feature selection with a 0.80 Pearson correlation threshold ensured low inter-feature correlations, enhancing robustness (Figure 7). This hybrid approach significantly improved performance, achieving an ACC of 97.48% and MCC of 95.01% in the full method (Table 2, D4). By integrating LightGBM’s non-linear modeling with shallow learning features, the resulting 4-dimensional vector provides a comprehensive and diverse input for TextCNN-based classification (Section 2.6), optimizing accuracy and generalization in plant-specific RBP prediction.

### 2.6. Deep Learning Method

To further enhance the feature extraction and classification capabilities for protein sequences, this study employs an improved Text Convolutional Neural Network (TextCNN) as the final classifier. TextCNN, originally proposed by Yoon Kim [46] in 2014 for text classification, applies CNN to sequential data, using multiple convolutional kernels of varying sizes (e.g., 3, 4, and 5) to extract local patterns, capturing spatial correlations within the sequence. This enables effective capture of spatial correlations within the sequence. With its simple architecture, fewer parameters, lower computational complexity, and faster training times compared to methods like LSTM, TextCNN is well-suited for large-scale sequence analysis. Given the sequential similarity between protein sequences and natural language, TextCNN has proven adaptable from natural language processing (NLP) [47] tasks to bioinformatics applications [48,49]. In this study, we introduce an optimized TextCNN by replacing its traditional word embedding layer with a custom sequence encoding method that integrates KPC features (*k* = 1, 2) and the prediction outputs from shallow and ensemble methods (as described in Section 2.4 and Section 2.5). This modification eliminates the need for generic embeddings, directly utilizing a 424-dimensional biologically relevant feature vector to improve classification performance for plant-specific RBPs. The model processes this input through convolution, global max pooling, and fully connected layers, achieving robust binary classification of RBPs versus non-RBPs.

### 2.7. Attention Mechanism

To enhance the discriminative capability of our method for plant-specific RBP prediction, we incorporate an attention mechanism into the TextCNN, enabling it to prioritize the most relevant features extracted from protein sequences. The attention mechanism, inspired by its success in natural language processing and sequence modeling, assigns weights to different feature representations based on their contextual importance, thereby focusing computational resources on biologically significant patterns. Following the methodology proposed by Vaswani et al. [50], we adopt an attention mechanism where the convolutional outputs from TextCNN—three 64-dimensional vectors derived from filter sizes 3, 4, and 5—are used as both query and value inputs. This self-attention process computes a weighted sum of the feature vectors, capturing dependencies across different convolutional scales and enhancing the model’s ability to interpret complex sequence patterns. The attention mechanism has been shown to improve classification performance in bioinformatics tasks, such as protein function prediction, by adaptively refining feature representations [51,52]. In this study, the attention layer integrates seamlessly with the 424-dimensional input vector, refining the feature set prior to final classification. By emphasizing critical sequence motifs associated with RNA-binding activity in plants, this mechanism contributes to the model’s superior accuracy and robustness.

In this study, we propose an improved TextCNN. We optimized the model structure by removing the word embedding layer and using the sequence encoding method introduced in this study directly as the input of the model. This improvement aims to fully utilize the feature expression ability of sequence encoding to improve the performance of the method in RBP prediction tasks while reducing the additional complexity introduced by the word embedding layer. Specifically, in the data input stage, each protein sequence in this study is first encoded into a fixed-length feature vector using the KPC, resulting in a vector of 424 dimensions (i.e., *n* = 424). For a dataset containing *m* protein sequences, this encoding process yields a two-dimensional matrix of shape *(m, n)*, where m represents the number of sequences and *n* denotes the encoded feature dimension. For the TextCNN, each encoded sequence is treated as a one-dimensional feature map with a length of *n* and a single channel, i.e., a tensor of shape (*n*, 1), analogous to a sequence of length *n* with a single feature channel. During the convolution stage, to effectively capture localized patterns within the encoded feature vector, this study employs three one-dimensional convolutional kernels with sizes 3, 4, and 5, respectively. Each kernel slides over the input feature map to extract abstract representations, focusing on the relationships between adjacent dimensions in the encoded vector. In the pooling stage, global max pooling is applied to each convolutional feature map to extract the most salient features, reducing the dimensionality while retaining the most significant information. Finally, in the fully connected stage, the outputs from all global max-pooling layers are concatenated to form a unified feature vector, which is subsequently used for the binary classification task of identifying RBPs.

### 2.8. Evaluation Metrics

In order to evaluate the performance and effectiveness of the method, we used common evaluation indicators to evaluate the predictive performance of the method. These evaluation indicators include ACC, MCC, ${F1}_{score}$, Sensitivity (SN), and Specificity (SP). The calculation of these indicators is shown in Equations (4) to (9).

(4) $$\begin{array}{c} ACC=\frac{TP+TN}{TP+FN+FP+FN}\times 100\% \end{array}$$

(5) $$\begin{array}{c} MCC=\frac{TP+TN+FP+FN}{\sqrt{\left(TP+FP\right)\times \left(TN+FN\right)\times \left(TP+FN\right)\times \left(TN+FP\right)}}\times 100\% \end{array}$$

(6) $$\begin{array}{c} SN=\frac{TP}{TP+FN}\times 100\% \end{array}$$

(7) $$\begin{array}{c} SP=\frac{TN}{TN+FP}\times 100\% \end{array}$$

(8) $$\begin{array}{c} {F1}_{score}=\frac{2\times TP}{2\times TP+FP+FN}\times 100\% \end{array}$$

(9) $$\begin{array}{c} Precision=\frac{TP}{TP+FP}\times 100\% \end{array}$$

where True Positive (TP) refers to the number of samples that are actually positive and correctly identified as such. True Negative (TN) indicates the number of samples that are actually negative and accurately predicted as negative. False Negative (FN) represents the number of samples that are actually positive but incorrectly classified as negative. False Positive (FP) denotes the number of samples that are actually negative but mistakenly predicted as positive.

## 3. Results and Discussion

In this section, to ensure that our method is not only stable but also advanced, we designed six experiments to comprehensively evaluate its performance. This study used Python 3.11 and PyCharm 2024.1 (JetBrains, Prague, Czech Republic) as experimental environments. The computer operating system is Windows Server 2019 (Microsoft Corporation, Redmond, WA, USA), equipped with an Intel(R) Xeon(R) CPU E5-1680 v4 @ 3.40 GHz (Intel Corporation, Santa Clara, CA, USA) and 64 GB of memory (Kingston Technology, Fountain Valley, CA, USA).

First, we verified the method using benchmark datasets and evaluated its performance with comprehensive metrics. Secondly, we explored the performance of the proposed method under different feature combinations and identified the features that contributed most to the performance of the method. This helps us understand which features are critical to improving prediction accuracy. Then, in the third stage, we conducted a comparative analysis of the performance of the machine learning methods used in this study. This step aims to show the advantages and limitations of different methods on specific tasks. In the fourth stage, we validated the performance of the method on the independent dataset. In the fifth stage, to further verify the effectiveness of our method, we compared its performance with other state-of-the-art methods. This comparison not only highlights the advantages of our method, but also provides a valuable reference for our future research direction. Through this series of experimental designs, our method has been rigorously tested and verified in multiple dimensions, thus ensuring its reliability and advancement in practical applications. Finally, we compared the proposed method with the original TextCNN method through experiments, further confirming its effectiveness.

### 3.1. Performance on Benchmark Dataset

In the first stage, we utilized 5-fold cross-validation to assess the performance of our proposed method on a benchmark dataset comprising 4992 protein sequences (2496 RBP and 2496 non-RBP sequences). Across the 5 folds, our method achieved excellent performance; the evaluation results are shown in Table 1. To ensure optimal convergence and prevent overfitting, early stopping was applied, halting training if the validation loss did not decrease for five consecutive epochs, with a maximum of 50 epochs. To further evaluate model stability, 10-fold cross-validation was conducted, and the results are provided in Supplementary Materials Table S1 and Figure S1.

Table 1 evaluates the performance of the proposed RBP prediction method using 5-fold cross-validation on a benchmark dataset, revealing consistently high performance across all folds. Fold 1 exhibits the lowest performance with 96.10% ACC, 98.89% AUC, and 92.27% MCC, while Fold 3 and Fold 5 achieve the highest ACC (97.60%), and Fold 2 achieves the second-highest SP (98.77%), after Fold 5 (99.43%). Across the folds, SN ranges from 94.27% to 96.68% and SP from 98.11% to 99.43%, demonstrating the method’s balanced capability to accurately identify both RBPs and non-RBPs.

**Figure 2:**
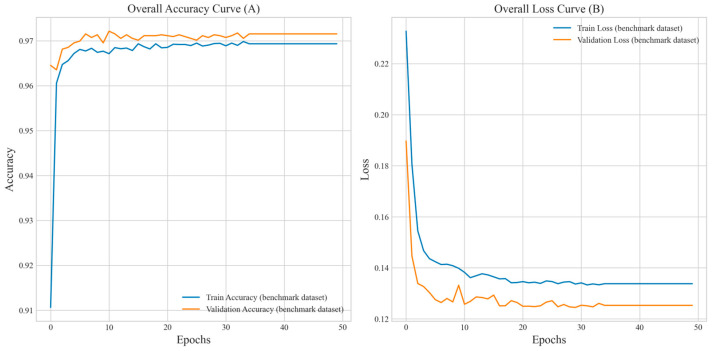
Shows the overall accuracy curve and loss curve for training, with the horizontal axis representing training epochs and the vertical axis indicating accuracy and loss, respectively. (**A**) demonstrates that the average accuracy of the training and validation sets rapidly increases in the initial stage, exceeding 0.97 after about 10 epochs, and then stabilizes with slight fluctuations, indicating that the method is approaching convergence on the benchmark dataset. (**B**) shows that the average loss values for both the training and validation sets rapidly decrease in the initial stage, stabilizing at around 0.13 after about 10 epochs. The validation and training set losses remain consistent, demonstrating good fitting performance, enhanced by early stopping to prevent overfitting, ensuring stable and robust performance. Overall, the method converges rapidly within 30 epochs, demonstrates excellent performance, and does not exhibit significant overfitting, making it suitable for application to this dataset.

To further assess the accuracy and loss dynamics of the method during 5-fold cross-validation, we present the accuracy and loss curves on the benchmark dataset, as illustrated in Figure 2.

These results indicate that the integration of KPC encoding with shallow and ensemble learning predictions effectively captures sequence patterns critical for RBP identification. The high ACC and MCC suggest strong predictive power, while the balanced SN and SP demonstrate the method’s ability to accurately classify both RBPs and non-RBPs.

### 3.2. Performance on Feature Combinations

**Figure 3:**
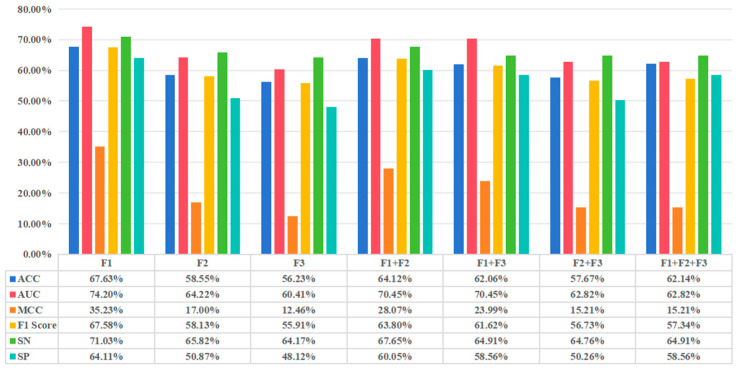
Presents the evaluation results of the method’s performance on the benchmark dataset using the KPC method, where F1, F2, and F3 represent the AAC, DPC, and tripeptide composition (TPC) features, respectively, for *k* = 1, 2, 3. The evaluation indicators include ACC, (MCC, ${F1}_{score}$, SN, and SP. When F1 is used alone, the model performs best, achieving an ACC of 67.63%, an MCC of 35.23%, and an ${F1}_{score}$ of 67.58%, indicating that the AAC feature has strong discriminative ability in capturing the sequence’s basic information. However, performance is poorest when F3 is used alone (ACC of 56.23%, MCC of 12.46%) due to its data sparsity caused by high dimensionality. In the feature combination, the ACC of F1 + F2 is 64.12%, slightly lower than F1, but also shows good balance, while the ACC of F1 + F2 + F3 (62.14%) is slightly lower than that of F1, indicating that feature stacking may introduce redundant information, weakening performance. With the introduction of F3, the encoding dimension increases to 8420 dimensions, requiring more computational resources.

In the second stage, we examined the performance of our proposed method using various features and feature combinations on the benchmark dataset under 5-fold cross-validation. We designate the features for k = 1 as F1, k = 2 as F2, and k = 3 as F3 in the KPC method. We evaluated the method’s performance using F1, F2, and F3 features and assessed the performance under various feature combinations. The results are presented in Figure 3.

**Figure 4:**
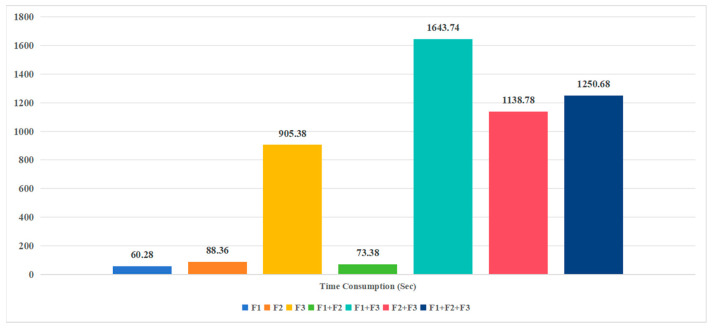
Illustrates the time consumption (in seconds) of different feature combinations used in the proposed RBP prediction model, with F1, F2, and F3. The results show that using F1 alone requires the least time at 60.28, followed by F2 at 88.36s, while F3 significantly increases the computational cost to 905.38s due to its higher dimensionality (8000 dimensions). Combining features further escalates the time: F1 + F2 takes 73.38s, F1 + F3 consumes 1643.74s, F2 + F3 requires 1138.78s, and the full combination F1 + F2 + F3 demands the time at 1250.68s. This indicates that while F1 and F2 offer a balance between computational efficiency and predictive performance (as shown in Figure 3), incorporating F3 substantially increases runtime, highlighting the trade-off between feature complexity and computational cost in RBP prediction tasks.

To further evaluate the computational efficiency of various feature combinations, this study presents the time consumption for each combination, as illustrated in Figure 4.

The superior performance of F1 alone suggests that AAC effectively captures basic sequence composition, while F3’s high dimensionality (8000 dimensions) introduces noise and sparsity, leading to suboptimal results. The combination of F1 and F2 balances global (amino acid frequency) and local (dipeptide dependency) sequence patterns, enhancing predictive accuracy and stability without excessive computational cost. However, the simplicity of F1 may result in the loss of complex sequence features. Therefore, we chose the combination of F1 and F2 with stronger representational ability and stability and further verified this in the experiment in Section 3.3.

### 3.3. Performance on Ensemble Learning Framework

**Figure 5:**
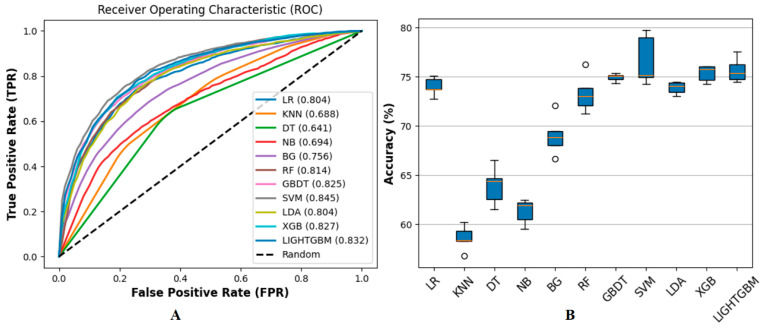
Illustrates the comparison of the ROC curves and ACC for the methods. This study compared the performance of 11 machine learning methods. (**A**) illustrates among these, SVM demonstrated the best performance with an AUC value of 0.845, and its ROC curve was closest to the upper left corner. LightGBM and XGB followed closely with AUC values of 0.832 and 0.827, respectively, while GBDT and RF also performed well with AUC values of 0.825 and 0.814. KNN, DT, and NB perform poorly, with AUC values below 0.700, specifically 0.688, 0.641, and 0.694, respectively. Overall, shallow learning methods such as SVM performed well, while LightGBM and XGB demonstrated advantages in classification ability and are suitable for complex data. In contrast, other methods like KNN and NB showed weaker performance and may require further optimization or replacement. (**B**) shows that SVM, GBDT, XGBoost, and LightGBM perform well in accuracy metrics, achieving an accuracy rate of over 75% and demonstrating excellent classification ability. The accuracy range for LR, RF, and LDA is 70% to 75%, indicating comparable performance. The accuracy of BG ranges from 65% to 70%, indicating a moderate level of performance. However, the accuracy of KNN, DT, and NB is relatively low, concentrated between 50% and 65%, reflecting poor performance.

In the third stage, we validated the performance of eleven different machine learning methods: LR, k-nearest neighbors (KNN) [53], decision tree (DT) [54], naive Bayes (NB) [55], bagging (BG) [56], RF, GBDT, SVM, LDA, XGBoost, and LightGBM on the benchmark dataset and evaluated their performance using AUC under 5-fold cross-validation, with ROC curves and ACC. In terms of feature combination, we used the feature combination of F1 and F2. The ROC curves illustrate significant performance differences among the methods, and we found that ensemble learning methods such as LightGBM, XGB, GBDT, and RF typically outperform other methods, indicating their robustness in capturing complex patterns within the data. We used the random parameter search method under 5-fold cross-validation for parameter optimization, and the optimized parameters refer to Supplementary Materials Table S2, and the results are shown in Figure 5.

The predictions derived from machine learning methods often encapsulate information inherent in the original feature set, potentially introducing significant correlation among features. To address this issue, we employed the Pearson correlation coefficient (r) [57] to perform a correlation analysis. This analysis is designed to detect and remove highly correlated features, thereby ensuring the independence and efficacy of the feature set and ultimately improving the method’s generalization performance and interpretability. The *r* can be defined as Equation (10).

(10) $$\begin{array}{c} r=\frac{Cov(x,y)}{\sigma x\cdot \sigma y} \end{array}$$

where Cov(x, y) represents the covariance of variables *x* and *y*, which *σx* and *σy* are the standard deviations of *x* and *y*, respectively.

**Figure 6:**
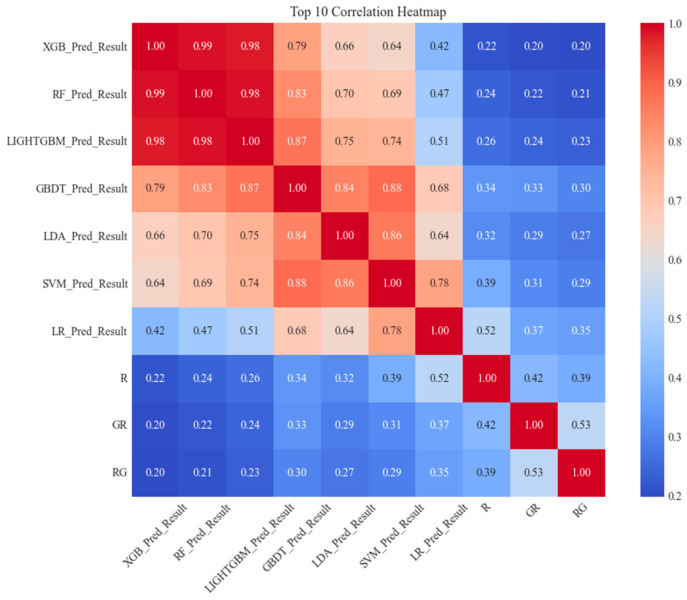
Shows a heatmap of the Pearson correlation coefficients between the prediction results of the machine learning models and the original features (R, GR, RG), reflecting the strength of the correlation between these predictions and the original features. The redder the color in the figure, the stronger the positive correlation between the features. The bluer the color, the stronger the negative correlation between the features. The heatmap indicates that the prediction results of XGBoost, RF, and LightGBM (XGB_Pred_Result, RF_Pred_Result, LIGHTGBM_Pred_Result) are highly correlated (0.98–0.99), suggesting that the predictions of these models are consistent and may capture similar patterns. In contrast, the prediction results of LR (LR_Pred_Result) show moderate correlation with the original features R, GR, and RG (0.35–0.52), and moderate to strong correlation with predictions from other models (0.42–0.78), indicating that LR may rely more on the original features due to its linear nature. Overall, the prediction results of the ensemble models are highly correlated with one another, likely due to shared feature representations, but weakly correlated with the original features (0.20–0.52).

Due to the high dimensionality of the features, we sorted them in descending order based on their correlation and provided a heatmap of the top 10 features with the highest correlation after sorting, as shown in Figure 6.

**Figure 7:**
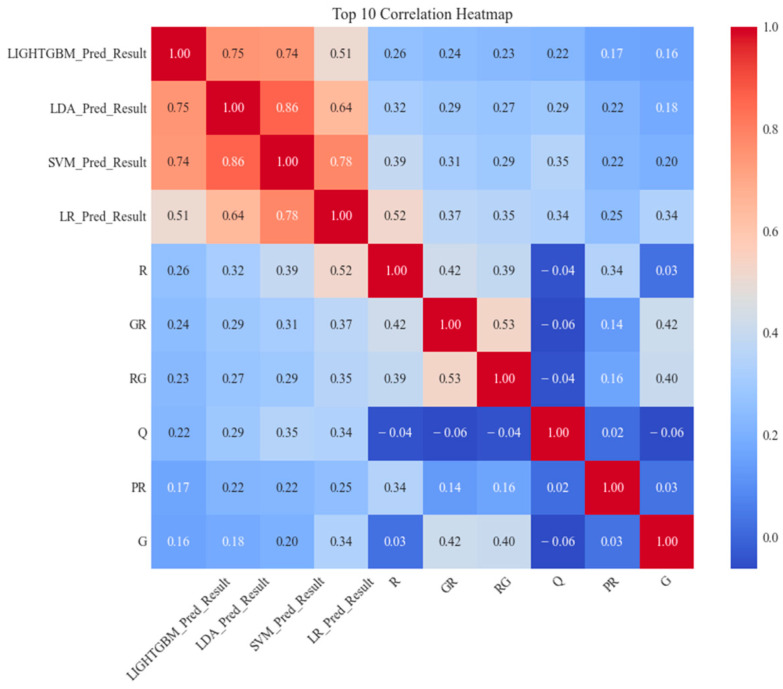
Shows that the correlation coefficient between LDA_Pred_Result and SVM_Pred_Result is 0.86, above the Pearson threshold of 0.80. We retained both due to their complementary mechanisms and high AUC values (0.845, 0.804). Other feature pairs, with correlations below 0.80 (0.20–0.79), were retained to ensure predictive diversity.

We used |r|≤0.80 as the threshold to screen and remove highly correlated features, as correlations above this value are typically considered high in machine learning tasks, potentially leading to multicollinearity [58]. For each pair of highly correlated features, we selected based on the AUC value of the ROC curve, retaining the features with higher AUC values and removing those with lower AUC values. For example, the correlation between the feature pairs XGB_Pred_Result and LightGBM_Pred_Result is 0.98, exceeding the set threshold. The AUC value for the XGB classifier is 0.827, while the AUC value for LightGBM is 0.832. Therefore, we removed XGB_Pred_Result from the feature set and retained LightGBM_Pred_Result to reduce feature redundancy and optimize method performance. Specifically, although the Pearson correlation coefficient between SVM_Pred_Result and LDA_Pred_Result is 0.86, exceeding the set threshold of |r|≤0.8, we still choose to preserve these two features based on the following considerations. First, there are essential differences in the prediction mechanisms of SVM and LDA (SVM processes non-linear data by maximizing the margin, while LDA performs linear discrimination based on inter-class variance), which may provide complementarity on different subsets of data. Second, the AUC values of the two are 0.845 and 0.804, respectively, indicating their significant contribution to classification performance. Removing any feature may result in information loss. After removing highly correlated features, we redrew the correlation heatmap, as shown in Figure 7.

**Figure 8:**
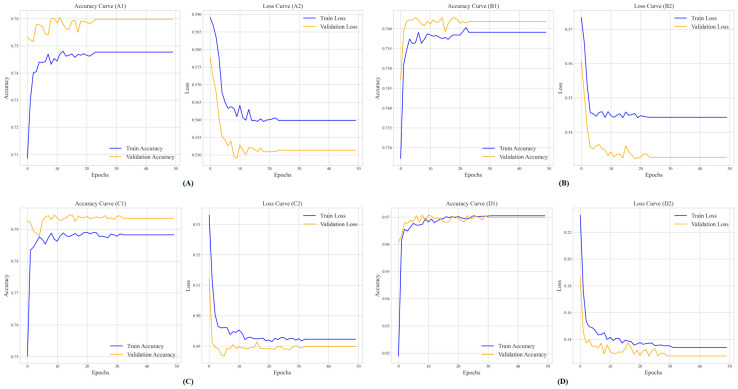
Illustrates the accuracy and loss curves of the proposed RBP prediction method. Subfigure (**A**) shows the average accuracy (**A1**) and loss (**A2**) curves of SVM, where training stops after about 20 epochs. The training accuracy was stable at around 0.750, while the validation accuracy fluctuated around 0.760. The loss decreased to between 0.550 and 0.565, indicating that early stopping alleviated overfitting despite the oscillation. Subfigure (**B**) shows the average accuracy (**B1**) and loss (**B2**) curves after adding LR, stopping after about 20 epochs. The training accuracy reached 0.758, the validation accuracy peak increased to 0.762, and the loss decreased to about 0.500, indicating an improvement compared to Subfigure (**A**) and showing that early stopping has better generalization ability. Subfigure (**C**) shows the average accuracy (**C1**) and loss (**C2**) curves with LDA added, stopping at 30 epochs. The training and validation accuracy converges between 0.780 and 0.800, while the loss stabilizes between 0.490 and 0.500, reflecting limited improvement that may be caused by feature redundancy. Finally, Subfigure (**D**) depicts the average accuracy (**D1**) and loss (**D2**) curves including LightGBM, which ends after 30 epochs. The training and validation accuracy converges to about 0.97, while the loss decreases to less than 0.14, demonstrating optimal convergence and stability and emphasizing the effectiveness of LightGBM and the benefits of early stopping in balancing performance and training efficiency.

In the final method, we chose three shallow learning methods, SVM, LR, and LDA, and one ensemble learning method, LightGBM, due to their ROC values exceeding 0.800, achieving good accuracy and meeting the correlation conditions we have set. We use the prediction results of these four methods as part of the feature set. Then, we added the predicted results one by one to the feature set and re-validated the model’s performance under identical TextCNN training (detailed parameters are shown in Supplementary Materials Table S3) using 5-fold cross-validation after each addition, and early stopping was applied when the validation loss did not decrease for 5 consecutive epochs, ensuring optimal convergence while preventing overfitting and the variation in stopping epochs reflects the differing convergence rates of the feature sets. Figure 8 shows the accuracy and loss curves after add different methods, respectively.

To further assess the performance improvements from ensemble learning, prediction results are evaluated under 10-fold cross-validation. We established a baseline TextCNN model using the F1 + F2 feature set (AAC and DPC, defined in Section 3.2), denoted as D0. We then incrementally enhanced the feature set, adding SVM predictions (D1), LR (D2), LDA (D3), and LightGBM (D4), with results in Table 2a,b and Figure 8.

Table 2a shows a clear upward trend across all performance metrics. The addition of SVM predictions (D1) significantly boosts performance, with ACC increasing from 64.24% (D0) to 76.28% (+12.04 percentage points), AUC from 70.42% to 82.61%, MCC from 28.02% to 53.36%, ${F1}_{score}$ from 64.02% to 76.08%, SN from 66.75% to 67.08%, and SP from 61.02% to 85.38%. Adding LR (D2) yields a smaller gain, with ACC rising to 76.68% (+0.40 percentage points), AUC to 83.74%, MCC to 53.55%, ${F1}_{score}$ to 76.62%, SN to 71.42%, and SP to 81.87%. Although the improvement from D1 to D2 is not statistically significant (*p* > 0.05, Table 2b), D2 was retained due to its contribution to model stability (lower standard deviation: ACC 1.28 vs. 1.35) and slight improvements in AUC (+1.13%) and SN (+4.34%), which support subsequent models. Notably, the inclusion of LDA (D3) further enhances performance, achieving an ACC of 79.41%, MCC of 58.89%, AUC of 86.40%, ${F1}_{score}$ of 79.39%, SN of 76.08%, and SP of 82.72%, improving the balance between sensitivity and specificity. This improvement aligns with LDA’s retention in the ensemble, directly resulting in increases from D2 to D3 in ACC (+2.73 percentage points), AUC (+2.66 percentage points), MCC (+5.34 percentage points), ${F1}_{score}$ (+2.77 percentage points), SN (+4.66 percentage points), and SP (+0.85 percentage points). The most significant improvement occurs with D4, where LightGBM’s inclusion drives ACC to 97.48%, AUC to 99.39%, MCC to 95.01%, ${F1}_{score}$ to 97.48%, SN to 95.66%, and SP to 99.31%, owing to LightGBM’s gradient boosting mechanism, which excels at handling complex feature interactions. This is further supported by Figure 8D, where D4 exhibits the fastest convergence and lowest validation loss, indicating enhanced training stability and robustness. Per-fold data are available in Supplementary Materials Table S4.

Table 2a demonstrates the progressive performance improvements across feature sets D0–D4, with D4 exhibiting superior metrics. To confirm the statistical significance of these differences, paired *t*-tests were conducted on the ACC and MCC of adjacent feature sets, as presented in Table 2b.

Table 2b shows *p*-values were calculated using paired *t*-tests (one-tailed, α = 0.05) based on per-fold ACC and MCC data from 10-fold cross-validation, comparing each feature set to the previous one; ‘vs.’ denotes comparison via paired *t*-tests. Shapiro–Wilk tests confirmed the normality of ACC and MCC data (*p*-values ranging from 0.236 to 0.944 for ACC and 0.276 to 0.978 for MCC), supporting the validity of *t*-tests. Detailed Shapiro–Wilk test results are provided in Supplementary Materials Table S5. The non-significant difference for D2 vs. D1 (*p* > 0.05) reflects the limited contribution of LR, but D2 was retained for its potential to enhance stability (lower standard deviation) and support subsequent models (D3, D4).

**Figure 9:**
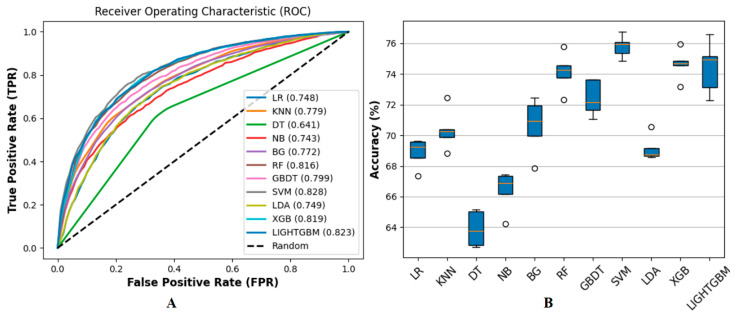
Shows the performance of 11 machine learning methods on the benchmark dataset under the F1 feature set (i.e., AAC, k = 1), which is evaluated by ROC curve (**A**) and accuracy (**B**). Compared with Figure 5A, the AUC values of all machine learning methods in (**A**) decreased; for example, LightGBM decreased from 0.832 to 0.823, and SVM decreased from 0.845 to 0.828. Similarly, (**B**) shows that the prediction accuracy is also generally reduced, with the highest accuracy of about 76%, which is about 2% lower than the highest value of 78% in Figure 5B. Specifically, the accuracy of each classifier is reduced; for example, LR is reduced from 74% to below 70%, and SVM is reduced from 79% to 76%. In addition, the prediction accuracy range of some classifiers has been widened, such as LightGBM and GBDT, indicating that the stability of the method has decreased [56,57].

Additionally, we have expanded the experimental in Section 3.2. We utilized F1 as a distinct feature set and presented the ROC curve and box plot of ACC under 5-fold cross-validation, as illustrated in Figure 9.

**Figure 10:**
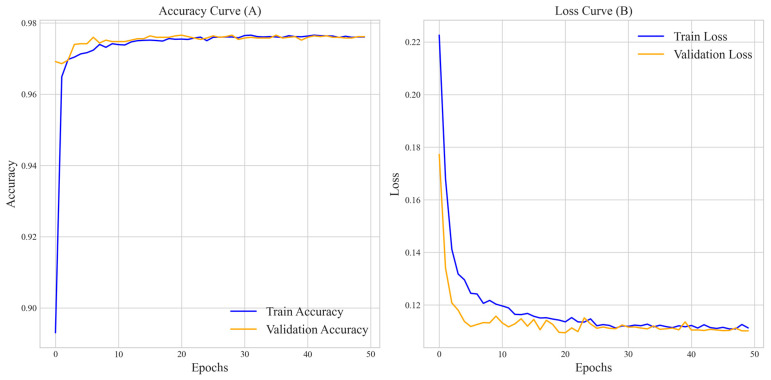
Illustrates the accuracy (**A**) and loss curves (**B**) of the method under the F1, utilizing early stopping and 5-fold cross-validation, while Figure 2A, B represents the same under the F1 + F2. In Figure 2A, the validation accuracy stabilizes at around 0.97 after 10 epochs, with losses dropping below 0.13 by 10 epochs, as seen in Figure 2B. In contrast, (**A**) shows that the validation set accuracy under F1 gradually stabilizes at 0.97 after 20 epochs. (**B**) shows a slower decrease in loss, with the validation set loss stabilizing at around 0.11 after 30 epochs. In addition, Figure 2 shows a more pronounced stability after stabilizing. This indicates that compared to F1 alone, the F1 + F2 combination improves convergence speed and stability by capturing global and local sequence patterns.

Furthermore, to evaluate the performance of the model using the F1 alone, we presented the accuracy and loss curves, as shown in Figure 10.

In summary, the experiment results indicate that the high AUC values of SVM (0.845) and LightGBM (0.832) highlight their effectiveness in capturing linear and non-linear sequence patterns, respectively. The retention of SVM, LR, LDA, and LightGBM after correlation-based feature selection (Pearson threshold of 0.80) ensures complementary feature contributions, as evidenced by the performance gains from D0 to D4 (Table 2). However, simpler methods like KNN and NB underperform due to their limited ability to handle high-dimensional sequence data, suggesting that ensemble methods are better suited for complex RBP prediction tasks.

### 3.4. Performance on Independent Dataset

In the fourth stage, we aimed to rigorously evaluate and validate the predictive performance of our proposed method for distinguishing RBPs from non-RBPs. To achieve this, we trained our method using a benchmark dataset comprising 4992 protein sequences (2496 RBPs and 2496 non-RBPs). This balanced training set was designed to ensure that the method could effectively learn the distinguishing features of both classes without bias toward either group, leveraging the diverse sequence characteristics captured in the dataset. The training process utilized KPC combined shallow learning methods, ensemble learning method, and TextCNN-based feature abstraction, as described earlier, to extract and process sequence-derived features effectively.

To assess the generalizability and robustness of our method, we validated the trained model on an independent dataset consisting of 1086 sequences (543 RBPs and 543 non-RBPs). The performance metrics obtained from this validation were excellent, demonstrating the efficacy of our method. Specifically, our method achieved an ACC of 99.72%, indicating an exceptionally high proportion of correct predictions across both classes. The ${F1}_{score}$, which balances precision and recall (SN), also reached 99.72%, underscoring the method’s consistency in identifying true positives while minimizing false positives and negatives. Furthermore, the MCC, a robust metric for binary classification that accounts for class imbalance and random guessing, was calculated at 99.45%, reflecting the near-perfect correlation between predicted and actual labels. SN, measuring the method’s ability to correctly identify RBPs, was 99.63%, while SP, indicating the accuracy in detecting non-RBPs, reached an impressive 99.82%. Here, ${F1}_{score}$ and precision were calculated using a weighted average (average = ‘weighted’) to account for the balanced dataset, resulting in a value close to ACC due to minimal misclassifications.

The performance on the independent dataset (ACC 99.72%, MCC 99.45%) demonstrates the method’s strong generalization to unseen data. The high SP (99.82%) indicates robust discrimination of non-RBPs. This performance validates the effectiveness of the KPC-based feature encoding and ensemble learning integration in capturing plant-specific RBP characteristics.

### 3.5. Comparison with State-of-the-Art Methods

In the fifth stage, to demonstrate the effectiveness of our proposed method for predicting plant-specific RBPs, we compare its performance with the RBPLight presented in [24], which has already been benchmarked against ten state-of-the-art RBP prediction tools using an independent dataset of 543 RBP and 543 non-RBP sequences. As reported in [24], among the existing tools, RBPPred achieved the highest ACC of 79.10%, with an ${F1}_{score}$ of 80.11% and an MCC of 58.50%, while Deep-RBPPred and IDRBP-ECHF exhibited the highest SN (85.08%) and SP (79.56%), respectively. In comparison, RBPLight outperformed all these tools, achieving an ACC of 86.74%, an ${F1}_{score}$ of 86.74%, an MCC of 73.48%, a precision of 86.74%, an SN of 86.74%, and an SP of 86.74%. Our method, significantly surpasses RBPLight on the same dataset, attaining an ACC of 99.72%, an ${F1}_{score}$ of 99.72%, an MCC of 99.45%, a precision of 99.72%, an SN of 99.63%, and an SP of 99.82%. This represents a substantial improvement over RBPLight, with an increase of 12.98 percentage points in both ACC, ${F1}_{score}$ and precision, and 25.97 percentage points in MCC, 12.89 percentage points in SN, and 13.08 percentage points in SP. Moreover, when compared to the best-performing tool (RBPPred) reported in [24], our method achieves a 20.62 percentage point higher ACC, a 19.61 percentage point higher ${F1}_{score}$, and a 40.95 percentage point higher MCC, underscoring its superior predictive capability. Additionally, our approach exhibits enhanced stability, with prediction intervals of approximately 75–78% for SVM, compared to 74–75% for GBDT, as inferred from their 5-fold cross-validation results. These results collectively demonstrate that our method not only outperforms RBPLight but also sets a new benchmark for plant-specific RBP prediction, surpassing all previously evaluated state-of-the-art tools reported in [24].

The substantial improvement over RBPLight (e.g., 12.98 percentage points in ACC) and other tools like RBPPred (20.62 percentage points in ACC) highlights the efficacy of our feature selection strategy, which mitigates redundancy (e.g., removing XGB_Pred_Result with a correlation of 0.98 to LightGBM_Pred_Result). This approach enhances generalization, making the method highly effective for plant-specific RBP prediction.

### 3.6. Comparison with Original TextCNN

To thoroughly assess the effectiveness of our improved TextCNN for predicting plant RBPs, we designed a comparative experiment against the original TextCNN, focusing on the impact of sequence encoding strategies on predictive performance. The original TextCNN employs a word embedding layer to transform protein sequences into numerical representations, which are processed through convolutional layers to automatically extract features. In contrast, our improved TextCNN replaces the word embedding layer with a manually designed sequence encoding method described in the previous sections. This method integrates a combination of sequence-based features, including AAC, DPC, and machine learning prediction result features (e.g., LightGBM_Pred_Result and SVM_Pred_Result), to capture information specific to plant RBPs. For the experiment, we utilized the same independent test dataset as in prior evaluations, consisting of 543 RBP and 543 non-RBP sequences, to ensure a fair comparison. The original TextCNN used the “one-hot” method for sequence encoding, which was input into the word embedding layer for semantic capture training, followed by convolution operations, pooling, and classification. Our improved TextCNN was trained under identical conditions but with the proposed sequence encoding method as input. Both models were evaluated using a comprehensive set of performance metrics, including accuracy, ${F1}_{score}$, MCC, SN, and SP. The results reveal that our improved TextCNN significantly outperforms the original across all metrics. Specifically, our method achieves an ACC of 99.72%, an ${F1}_{score}$ of 99.72%, an MCC of 99.45%, a precision of 99.72%, an SN of 99.63%, and an SP of 99.82%. In comparison, the original TextCNN yields an ACC of 74.49%, an ${F1}_{score}$ of 74.49%, an MCC of 48.99%, a precision of 74.50%, an SN of 74.03%, and an SP of 74.95%. This corresponds to a 25.23% increase in ACC, a 25.23% increase in ${F1}_{score}$, and a 50.46 percentage point increase in MCC, underscoring the superiority of our method. Furthermore, our improved TextCNN demonstrates higher performance.

The 25.23% increase in ACC over the original TextCNN underscores the advantage of replacing generic word embeddings with manually designed features (AAC, DPC, and machine learning predictions). These features capture biologically relevant sequence patterns, enhancing predictive accuracy. While deep learning excels at automatic feature extraction, our results suggest that domain-specific feature engineering remains critical for specialized bioinformatics tasks like plant RBP prediction.

In this study, we developed an innovative ensemble learning framework for predicting plant-specific RBPs, seamlessly integrating shallow and deep learning techniques to achieve state-of-the-art performance. The integration of KPC encoding (k = 1, 2) with predictions from SVM, LR, LDA, and LightGBM provides a robust feature set, as evidenced by the significant performance gains from D0 (ACC 64.24%) to D4 (ACC 97.48%) in Table 2. Unlike RBPLight, which relies on computationally intensive PSSM-derived features, our method uses lightweight sequence-based encoding, reducing computational cost (e.g., 73.38 s for F1 + F2) while achieving a 12.98 percentage point higher ACC on the independent test dataset. The Pearson correlation-based feature selection (threshold 0.80) further enhances generalization by mitigating redundancy, as seen in the removal of XGB_Pred_Result (correlation 0.98 with LightGBM_Pred_Result).

However, our method’s reliance on sequence-based features may limit its ability to capture structural or physicochemical properties, which could be critical for RBPs with complex binding mechanisms. For example, RBPPred incorporates dihedral angles and physicochemical properties, potentially improving predictions for structurally diverse RBPs. Additionally, this study does not account for longer sequence motifs (>3 amino acids) or spatial structural features due to the limited availability of comprehensive structural data for plant-specific RBPs in the CISBP-RNA and UniProtKB datasets. Inverse problem experiments, such as validating predictions against known structural interactions of proteins like Argonaute, which mediate RNA silencing in plants, such experiments could enhance the reliability of sequence-based predictions by cross-referencing them with experimentally determined RNA-binding interfaces. Moreover, our model demonstrated high prediction credibility, achieving 97.20% (5-fold) and 97.06% (10-fold) ACC on the benchmark dataset and 99.72% ACC (MCC 99.45%) on the independent test dataset. The ~2.5% performance gap likely arises from data noise in the larger, more diverse benchmark dataset (4992 sequences) or KPC encoding’s reliance on short motifs (k = 1,2), which may miss complex patterns. The high dimensionality of F3 (8000 dimensions) led to performance degradation, suggesting that overly complex features introduce noise rather than predictive power. To address these limitations, future work could leverage AlphaFold-predicted 3D structures to model RBP/RNA interactions and validate predictions using well-characterized RBPs, such as Argonaute proteins. Meanwhile, incorporate longer motifs or noise reduction techniques to enhance stability across datasets. Additionally, refining the negative dataset to include functionally similar proteins, such as transcription factors and DNA-binding proteins, could enhance the model’s specificity by better challenging its ability to distinguish RBPs from non-RBPs with overlapping functional roles. This approach would likely reduce false positives, improving the model’s robustness in complex biological scenarios. Extending the method to multi-label RBP classification or cross-species applications (e.g., human or bacterial RBPs) could further broaden its impact, providing deeper insights into RBP functions across diverse biological systems. Our approach offers a scalable solution for large-scale RBP annotation, with potential applications in identifying stress-response RBPs for crop breeding.

## 4. Conclusions

This study highlights the effectiveness of integrating shallow, ensemble and deep learning techniques for predicting plant-specific RBPs, achieving substantial performance improvements over existing methods. The use of KPC with k = 1 and k = 2 proved effective in capturing both global and local sequence patterns, providing a robust foundation for feature representation. The integration of prediction results from LR, SVM, LDA, and LightGBM as features enabled our improved TextCNN to discern intricate biological patterns critical for accurate RBP prediction. Careful feature selection, using a Pearson correlation threshold of 0.80, significantly enhanced generalization, as evidenced by performance gains from D0 to D4 (e.g., ACC increased from 64.24% to 97.48%). Compared to RBPLight, our method’s lightweight sequence-based encoding offers computational efficiency and superior accuracy (12.98 percentage points higher ACC on the independent test set). The 25.23 percentage point ACC improvement over the original TextCNN validates the advantage of domain-specific feature engineering. Future research could explore hybrid feature sets combining sequence, structural, and physicochemical properties or optimize the method with lightweight architectures to enhance scalability. This method provides an powerful tool for large-scale RBP annotation, supporting applications in stress-resistant crop breeding and advancing plant genomics research.

## Figures and Tables

**Table 1:** Performance of 5-fold cross-validation method.

Folds	ACC (%)	AUC (%)	MCC (%)	${F1}_{score}$ (%)	SN (%)	SP (%)
Fold 1	96.10	98.89	92.27	96.10	94.27	98.11
Fold 2	97.30	99.42	94.64	97.30	95.90	98.77
Fold 3	97.60	99.38	95.19	97.59	96.68	98.45
Fold 4	97.39	99.57	94.82	97.39	96.22	98.59
Fold 5	97.60	99.19	95.23	97.59	95.59	99.43
Average	97.20 ± 0.56	99.29 ± 0.23	94.43 ± 1.10	97.19 ± 0.56	95.73 ± 0.82	98.67 ± 0.44

**Table 2:** (**a**) Performance of different feature sets. (**b**) Statistical significance of performance differences between adjacent feature sets.

(**a**)						
**Feature Set**	**ACC (%)**	**AUC (%)**	**MCC (%)**	${F1}_{score}$ **(%)**	**SN (%)**	**SP (%)**
D0	64.24 ± 2.26	70.42 ± 3.14	28.02 ± 4.94	64.02 ± 2.53	66.75 ± 5.43	61.02 ± 8.97
D1	76.28 ± 1.35	82.61 ± 1.78	53.36 ± 2.82	76.08 ± 1.34	67.08 ± 2.04	85.38 ± 2.11
D2	76.68 ± 1.28	83.74 ± 1.53	53.55 ± 2.57	76.62 ± 1.29	71.42 ± 2.21	81.87 ± 1.55
D3	79.41 ± 1.79	86.40 ± 1.73	58.89 ± 3.58	79.39 ± 1.80	76.08 ± 2.05	82.72 ± 2.36
D4	97.48 ± 0.83	99.39 ± 0.23	95.01 ± 1.62	97.48 ± 0.83	95.66 ± 1.34	99.31 ± 0.57
(**b**)						
**Feature Set Comparison**			***p*** **-Value (ACC)**		***p*** **-Value (MCC)**	
D1 vs. D0			<0.00001		<0.00001	
D2 vs. D1			0.16304		0.3848	
D3 vs. D2			0.00034		0.00022	
D4 vs. D3			<0.00001		<0.00001	

## Data Availability

The source code and datasets for the LMFE project are hosted on GitHub and can be accessed at https://github.com/MPU-Ben/PlantRBPPrediction, accessed on 18 May 2025.

## Supplementary Materials

The following supporting information can be downloaded at https://www.mdpi.com/article/10.3390/biology14060672/s1, Table S1: Performance of 10-fold Cross-Validation Method. Table S2: Hyperparameter Ranges and Optimal Values of Machine Learning Methods. Table S3: TextCNN Model Parameters. Table S4: Detailed Per-Fold Data for All Feature Sets and Metrics. Table S5: Shapiro–Wilk Normality Test Results for ACC and MCC. Figure S1: Accuracy and Loss Curve Under 10-Fold Cross-Validation.

## Abbreviations

The following abbreviations are used in this manuscript:

RBPs	RNA-Binding Proteins
GO	Gene Ontology
PSSM	Position-Specific Scoring Matrix
KPC	K-Peptide Composition
AAC	Amino Acid Composition
DPC	Di-Peptide Composition
CD-HIT	Cluster Database at High Identity with Tolerance
SVM	Support Vector Machine
LR	Logistic Regression
LDA	Linear Discriminant Analysis
LightGBM	Light Gradient Boosting Machine
TextCNN	Text Convolutional Neural Network
CNN	Convolutional Neural Network
RF	Random Forest
GBDT	Gradient Boosting Decision Tree
XGB	Extreme Gradient Boosting
KNN	K-Nearest Neighbors
DT	Decision Tree
NB	Naive Bayes
BG	Bagging
ACC	Accuracy
AUC	Area Under Curve
MCC	Matthews Correlation Coefficient
SN	Sensitivity
SP	Specificity
XGB-VIM	XGB Variable Importance Measures
LGBM-VIM	LightGBM Variable Importance Measure
