# Supplementary material for: Research on Plant RNA-Binding Protein Prediction Method Based on Improved Ensemble Learning
Source: Biology (Basel). 2025 Jun 10;14(6):672. doi: 10.3390/biology14060672 (PMC12189372; doi:10.3390/biology14060672)
Supplement: Supplementary file 1 [file biology-14-00672-s001.zip › biology-3615828-supplementary.pdf]

Supplementary Table S1. Performance of 10-Fold Cross Validation.

Folds	ACC (%)	AUC (%)	MCC (%)	$F1_{score}$ (%)	SN (%)	SP (%)
Fold 1	95.80	98.86	91.66	95.81	93.91	98.19
Fold 2	95.60	99.03	91.38	95.59	92.28	98.82
Fold 3	98.40	99.39	96.81	98.40	97.57	99.21
Fold 4	96.99	99.40	94.07	97.00	95.08	99.15
Fold 5	97.19	99.46	94.42	97.19	95.98	98.40
Fold 6	97.80	99.45	95.62	97.79	95.71	99.62
Fold 7	96.59	99.25	93.28	96.59	94.62	98.74
Fold 8	97.80	99.63	95.62	97.79	96.28	99.22
Fold 9	97.80	99.08	95.65	97.79	96.00	99.60
Fold 10	96.59	99.16	93.22	96.58	93.36	99.27
Average	97.06±0.87	99.27±0.22	94.17±1.71	97.05±0.87	95.08±1.49	99.02±0.45

Table S1 evaluates the performance of the proposed RBP prediction method using 10-fold cross-validation on a benchmark dataset, demonstrating high consistency across all folds. Fold 2 exhibits the lowest performance with an ACC of 95.60% and MCC of 91.38%, while Fold 3 achieves the highest ACC (98.40%) and MCC (96.81%). Across the folds, SN ranges from 92.28% to 97.57%, and SP varies from 98.19% to 99.62%, reflecting the method's robust ability to accurately classify both RBPs and non-RBPs. These results are comparable to the 5-fold cross-validation (Table 1), further validating the method's reliability, with LightGBM 's contribution to feature integration enhancing performance.

Supplementary Table S2. Hyperparameter Ranges and Optimal Values of Machine Learning Methods.

Methods	Hyperparameter	Predefined Range	Optimal Value
LR	C	[0.001, 0.01, 0.1, 1, 10, 100]	100
	solver	['newton-cg', 'lbfgs', 'liblinear', 'sag', 'saga']	liblinear
KNN	weights	['uniform', 'distance']	distance
	n_neighbors	[3, 5, 7, 9, 11]	3
DT	max_depth	None, 10, 20, 30	None
	min_samples_split	[2, 5, 10]	2
SVM	C	[0.1, 1, 10, 100]	1
	kernel	['linear', 'rbf', 'poly']	rbf
	gamma	['scale', 'auto']	scale
LDA	solver	['svd', 'lsqr', 'eigen']	svd
	priors	[None, [0.5, 0.5]]	None
RF	n_estimators	[50, 100, 200]	200
	max_depth	[None, 10, 20]	10
	min_samples_split	[2, 5, 10]	2
GBDT	n_estimators	[50, 100, 200]	200
	learning_rate	[0.01, 0.1, 0.2]	0.1
	max_depth	[3, 5, 7]	7
XGB	n_estimators	[50, 100, 200]	200
	learning_rate	[0.01, 0.1, 0.2]	0.2
	max_depth	[3, 5, 7]	5
	subsample	[0.6, 0.8, 1.0]	0.8
BG	n_estimators	[10, 50, 100]	100
	max_samples	[0.5, 0.75, 1.0]	0.75
	max_features	[0.5, 0.75, 1.0]	0.5
	bootstrap	[True, False]	False
LightGBM	n_estimators	[50, 100, 200]	200
	learning_rate	[0.01, 0.1, 0.2]	0.2
	max_depth	[3, 5, 7]	5
	num_leaves	[31, 63, 127]	63
GaussianNB	priors	[0.7, 0.3, None]-	None
	var_smoothing	np.logspace (0, -9, num=100)	1e-06

Table S2 shows the optimized parameters and their corresponding values for each model in this research.

Supplementary Table S3. TextCNN Model Parameters.

Parameters	Value
filter_sizes	[3,4,5]
num_filters	64
num_classes	2
feature_dim	424
epochs	50
batch_size	32
dropout_rate	0.5
L2_reg_lambda	0.001
optimizer	Adam (learning rate = 0.001)
callback	EarlyStopping (monitor='val_loss', patience=5, restore_best_weights=True)

Table S3 shows the optimized parameters and their corresponding values for each model in this research. Here, "-" denotes has no parameters to optimize.

Supplementary Table S4. Detailed Per-Fold Data for All Feature Sets and Metrics.

Feature Set	Folds	ACC (%)	AUC (%)	MCC (%)	F1score (%)	SN (%)	SP (%)
D0	Fold 1	61.20	65.29	19.45	59.50	78.85	38.91
	Fold 2	61.20	67.47	22.53	61.17	64.23	58.27
	Fold 3	64.53	69.01	29.05	64.53	64.37	64.68
	Fold 4	63.93	73.46	27.29	63.58	72.73	54.04
	Fold 5	66.33	73.90	32.78	66.28	70.28	62.40
	Fold 6	64.33	70.72	28.15	64.25	59.23	68.8
	Fold 7	62.32	67.84	24.41	62.28	65.77	58.58
	Fold 8	63.53	68.14	27.01	63.53	62.81	64.20
	Fold 9	68.54	74.61	37.12	68.52	66.40	70.68
	Fold 10	66.53	73.72	32.44	66.53	62.83	69.6
D1	Fold 1	75.8	81.93	53.06	75.84	69.53	83.71
	Fold 2	74.6	80.45	49.67	74.41	66.26	82.68
	Fold 3	78.36	85.14	57.71	78.13	68.42	88.1
	Fold 4	76.35	84.17	54.07	76.27	68.56	85.11
	Fold 5	76.55	85.54	53.90	76.37	67.87	85.20
	Fold 6	78.16	82.83	56.37	77.90	68.24	86.84
	Fold 7	75.55	82.46	52.01	75.48	68.85	82.85
	Fold 8	73.95	79.77	48.53	73.60	62.81	84.44
	Fold 9	77.35	81.84	56.44	77.02	65.2	89.56
	Fold 10	76.15	81.96	51.82	75.82	65.04	85.35
D2	Fold 1	75.8	83.11	52.35	75.88	71.68	81.00
	Fold 2	76.00	81.68	52.23	75.90	69.92	81.89
	Fold 3	78.16	85.74	56.51	78.10	73.28	82.94
	Fold 4	78.36	85.10	57.14	78.36	74.62	82.55
	Fold 5	76.75	85.95	53.71	76.71	72.29	81.20
	Fold 6	77.15	83.98	54.14	76.98	69.10	84.21
	Fold 7	75.55	83.19	51.35	75.55	72.69	78.66
	Fold 8	74.15	81.06	48.47	73.98	66.53	81.32
	Fold 9	78.16	84.26	56.71	78.08	72.4	83.94
	Fold 10	76.75	83.37	52.94	76.69	71.68	80.95
D3	Fold 1	79.40	86.21	58.78	79.46	78.14	81.00
	Fold 2	76.40	82.71	52.96	76.33	71.14	81.50
	Fold 3	80.76	87.26	61.57	80.75	78.14	83.33
	Fold 4	80.96	88.14	62.63	80.95	75.76	86.81
	Fold 5	80.36	89.36	60.84	80.34	77.11	83.60
	Fold 6	82.36	87.39	64.55	82.32	78.11	86.09
	Fold 7	77.56	85.55	55.27	77.56	75.38	79.92
	Fold 8	77.15	85.05	54.25	77.14	75.21	78.99
	Fold 9	78.96	86.30	58.13	78.92	74.8	83.13
	Fold 10	80.16	85.99	59.90	80.14	76.99	82.78

D4	Fold 1	96.00	98.93	92.14	96.01	93.55	99.10
	Fold 2	96.00	99.06	92.14	96.00	93.09	98.82
	Fold 3	98.40	99.55	96.82	98.40	97.17	99.60
	Fold 4	97.39	99.58	94.84	97.40	95.83	99.15
	Fold 5	97.39	99.52	94.80	97.39	96.79	98.00
	Fold 6	98.60	99.58	97.22	98.60	97.00	100.00
	Fold 7	97.60	99.50	95.27	97.60	95.77	99.58
	Fold 8	97.80	99.65	95.67	97.79	95.45	100.00
	Fold 9	98.00	99.28	96.02	98.00	96.80	99.20
	Fold 10	97.60	99.24	95.21	97.59	95.13	99.63

Table S4 shows the detailed per-fold results for D4 from the same 10-fold cross-validation run as Table S1, with minor variations due to early stopping.

Supplementary Table S5. Shapiro-Wilk Normality Test Results for ACC and MCC.

FeatureSet	ACCW	ACCp-value	MCCW	MCCp-value
D0	0.944	0.586	0.963	0.790
D1	0.974	0.914	0.960	0.761
D2	0.961	0.767	0.962	0.780
D3	0.947	0.622	0.959	0.747
D4	0.927	0.418	0.946	0.606

Table S5 shows Shapiro-Wilk Normality Test Results for ACC and MCC from 10-Fold Cross Validation. (ACCW and MCCW: Shapiro-Wilk test statistic W; ACCp-value and MCCp-value: corresponding p-values; $p>0.05$ indicates normality.)

Supplementary Figure S1. Accuracy and Loss Curve Under 10-Fold Cross Validation.

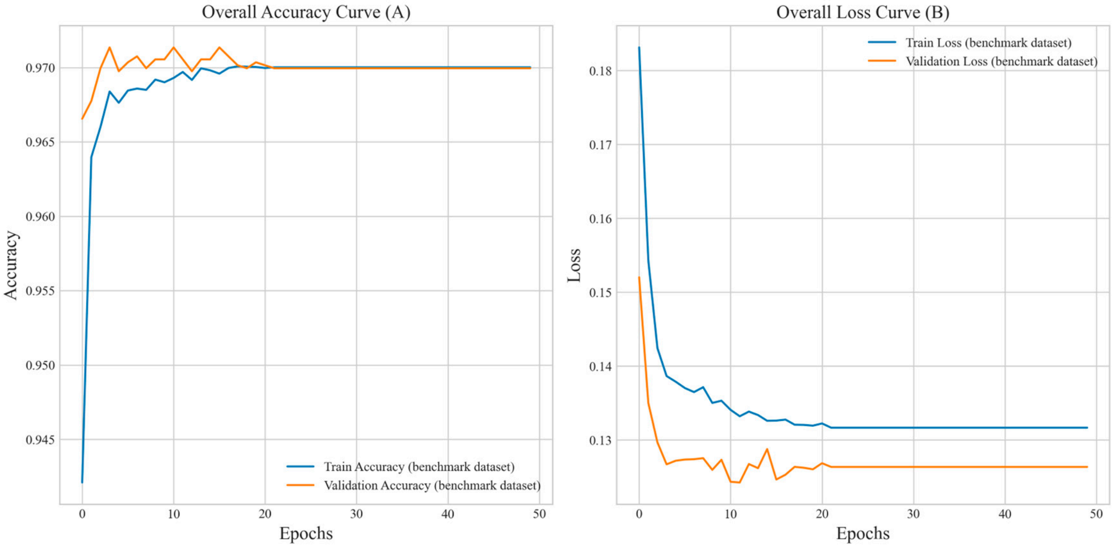

Figure S1 presents the overall accuracy (A) and loss (B) curves of the proposed method under 10-fold cross-validation on the benchmark dataset across 50 epochs, highlighting the method's training dynamics. (A) shows that the training accuracy rapidly increases to around 0.970 within the first 10 epochs and stabilizes near 0.972, while the average validation accuracy rises to approximately 0.973. Subsequently, there was slight oscillation within the range of 20 epochs and it stabilized at this accuracy. (B) indicates that within 20 epochs, the training loss decreased from 0.19 to below 0.14 and remained stable thereafter, while the validation loss decreased to around 0.13 and remained stable. These trends are consistent with the average performance metrics reported in Supplementary Table S1 (97.24% ACC, 99.22% AUC), emphasizing the robust convergence and generalization ability of the method, and enhanced by the feature ensemble of LightGBM**.**
